# Vasculogenic potential of adipose tissue derived stem cells from patients with chronic spinal cord injury and pressure injuries

**DOI:** 10.1007/s10456-025-10002-y

**Published:** 2025-09-10

**Authors:** Ángela Santos-De-La-Mata, Pedro F. Esteban, Mario Martínez-Torija, Beatriz Paniagua-Torija, Fa. Javier Espino-Rodríguez, Lucía Beltrán-Camacho, Celia Camacho-Toledano, Mónica Alcobendas-Maestro, Fernando García-García, Eduardo Molina-Holgado, Ma Carmen Durán-Ruiz, Juan M. Melero-Martin, Rafael Moreno-Luna

**Affiliations:** 1 Pathophysiology and Regenerative Medicine Group, Hospital Nacional de Parapléjicos, Servicio de Salud de Castilla la Mancha (SESCAM), 45071 Toledo, Spain; 2 Pathophysiology and Regenerative Medicine, Instituto de Investigación Sanitaria de Castilla-La Mancha (IDISCAM), Toledo, Spain; 3 Grupo de Neuroinflamación, Hospital Nacional de Parapléjicos, SESCAM, Toledo, Spain; 4 Grupo de Neuroinflamación, Instituto de Investigación Sanitaria de Castilla-La Mancha (IDISCAM), Toledo, Spain; 5 Plastic and Reconstructive Surgery Service, Hospital Nacional de Parapléjicos, SESCAM, Toledo, Spain; 6 Biomedicine, Biotechnology and Public Health Department, Science Faculty, Cádiz University, 11002 Cádiz, Spain; 7 Department of Physical Rehabilitation, Hospital Nacional de Paraplejicos, SESCAM, 45071 Toledo, Spain; 8 Radiodiagnostic Service, Hospital Nacional de Parapléjicos, SESCAM, Toledo, Spain; 9 Biomedical Research and Innovation Institute of Cadiz (INiBICA), Cadiz, Spain; 10 Department of Cardiac Surgery, Boston Children’s Hospital, Boston, MA 02115 USA; 11 Department of Surgery, Harvard Medical School, Boston, MA 02115 USA; 12 Harvard Stem Cell Institute, Cambridge, MA 02138 USA

**Keywords:** Spinal cord injury, Pressure injury, Adipose tissue, Angiogenesis, Vasculogenesis, ASCs, MSCs, ECFCs

## Abstract

Limited vascularization and ischemia are major contributors to the chronicity of wounds, such as ulcers and traumatic injuries, which impose significant medical, social, and economic burdens. These challenges are particularly pronounced in patients with spinal cord injury (SCI), a disabling condition associated with vascular dysfunction, infections, and impaired peripheral circulation, complicating the treatment of pressure injuries (PIs) and the success of reconstructive procedures like grafts and flaps. Regenerative medicine aims to address these issues by identifying effective cellular therapies to restore vascular beds. Among these, cells from the stromal vascular fraction (SVF) of adipose tissue (AT) are promising due to their abundance of angiogenic and vasculogenic cells, including mesenchymal stem cells (MSCs) and endothelial colony-forming cells (ECFCs). This study evaluated the vasculogenic potential of AT-derived cellular fractions isolated via enzymatic digestion of white adipose tissue (WAT). We compared adipose-derived stem cells (ASCs) cultured from SVF with a combination of ECFCs and MSCs, expanded separately and transplanted in a 40:60 ratio. Results showed that while ASCs promote angiogenesis and vasculogenesis, the ECFC/MSC combination is superior, consistently forming perfused vascular beds in subcutaneous implants in nude mice. Furthermore, ECFCs and MSCs extracted from small amounts of WAT in SCI patients with PIs demonstrated similar functionality and vasculogenic potential to cells from healthy controls. These findings highlight the potential of AT-derived ECFCs and MSCs in autologous cell therapies, offering a promising avenue for advancing vascular regeneration in patients with SCI.

**Supplementary Information:**

The online version contains supplementary material available at 10.1007/s10456-025-10002-y.

## Introduction

An inadequate blood supply and the resulting tissue ischemia play a pivotal role in the persistence and chronic nature of wounds such as ulcers, burns, and traumatic injuries [1]. These wounds often fail to heal properly, leading to prolonged tissue damage, increased susceptibility to complications, and significant social and economic costs [2, 3]. Among the most affected populations, individuals with spinal cord injury (SCI) are particularly vulnerable. As a permanent incapacitating syndrome, SCI remains a global health challenge, affecting millions of individuals worldwide with enduring disabilities [4, 5]. Beyond the well-documented motor and sensory impairments, SCI is acknowledged as a complex clinical condition that not only predisposes patients to pressure injuries (PIs) [6], but also exacerbates their severity due to an elevated risk of vascular dysfunction, recurrent infections, and compromised peripheral circulation [4, 5, 7–9], what significantly impacts the health of SCI patients.

PIs and other chronic wounds represent a substantial source of morbidity and mortality in SCI patients, besides placing a heavy financial burden on healthcare systems. In developed countries, the management of PIs alone can account for up to 4% of annual medical expenses [10, 11]. For instance, at the Hospital Nacional de Parapléjicos (HNP) in Spain, more than 149 SCI patients with advanced-stage PIs undergo treatment each year, often requiring surgical interventions that extend their hospital stays and contribute to healthcare costs totaling approximately 4.1 million euros annually. Despite these efforts, surgical interventions are not always effective, leaving many patients without satisfactory solutions and underscoring the need for innovative approaches to address the unique challenges of chronic wounds [12].

Modern medicine has started exploring alternatives to standard practices for repairing and rebuilding tissues, especially when protocolized surgical procedures have proven ineffective [13]. Among the different approaches, the use of adipose tissue (AT) has gained interest due to the presence of various cell types with regenerative potential [14]. The AT-derived stromal vascular fraction (SVF) harbors a heterogeneous assembly of cells, notably enriched with mesenchymal stem cells (MSCs), rendering it a compelling candidate for cell therapy due to its autologous nature and suitability for point-of-care application [15]. Nevertheless, the presence of a diverse cell population within the SVF has traditionally been overlooked in cell-based therapies [16]. This oversight poses significant challenges for conducting efficacy and safety validation studies, as the lack of standardized cell counts and concentrations impedes reliable assessments. Consequently, the efficacy of this therapeutic approach may exhibit considerable variability among patients [17].

Alternatively, adipose stem cells (ASCs) [18], also known as adipose-derived MSCs [19] are one of the most promising sources of stem cell populations with significant potential in clinical medicine [20], particularly in the field of neovascularization [21]. These cells can be obtained as result of culturing SVF in vitro [22]. Indeed, the adherence and proliferation capacity of some cell populations within the SVF facilitate the obtention of a more homogeneous and stable population of ASCs rich in MSCs [23]. MSCs have demonstrated multipotent differentiation capacity, including the potential to differentiate into perivascular cells [24, 25], as well as immunomodulatory properties, promoting allograft survival and tolerance in cell-therapy studies [24]. Besides, other findings suggest that their efficacy is enhanced when co-administered with endothelial cells (ECs) capable of de novo vascular bed regeneration [26, 27].

Endothelial colony-forming cells (ECFCs) comprise a type of progenitor cells identified in various tissues, including blood, endothelial lining, and adipose tissue [28]. However, their low abundance in circulation makes their purification unfeasible in many patients, with subcutaneous WAT being the most viable source [28]. ECFCs are ECs with clonal ability, capable of completing or forming new blood vessels [29], which are believed to play a crucial role in vascular repair following injury [30, 31]. Currently, researchers are exploring the therapeutic potential of ECFCs for the repair or regeneration of different tissues [32, 33].

Herein, we have evaluated the vasculogenic potential of different cellular combinations derived from SVF directly isolated from white adipose tissue (WAT). Moreover, we have explored the potential use of these cells as an alternative for revascularization in SCI patients. Our data suggests that the combination of ECFCs and MSCs extracted from the SVF of WAT is more effective for implant revascularization than the use of ASCs derived from the same SVF. Furthermore, we demonstrated that these cells, isolated from SCI patients, maintain the capacity to form functional blood vessels in vivo, emphasizing their potential for vascular repair.

## Materials and methods

### Study population

In this study, we enrolled SCI patients with undergoing reconstructive surgery for PIs at the Hospital Nacional de Parapléjicos (HNP), as well as patients without SCI or PIs undergoing general surgeries at hospitals within the Complejo Hospitalario de Toledo, Toledo, Spain. The study received approval from the local Institutional Review Board (Ethical and Clinical Research Committee of the Toledo Hospital Complex, code 207, approval date: 02/14/2018), and it adhered to the principles outlined in the Declaration of Helsinki. Additionally, compliance with Spanish law (Organic Data Protection Law 3/2018) was ensured to safeguard personal data protection. All patients provided informed consent prior to their inclusion in the study.

### Study design

This study aims to identify the most effective adipose tissue-derived cell population for vascular bed formation, with the goal of selecting the optimal candidate for potential cell-based therapies. Specifically, we investigate whether these cells remain viable when isolated from individuals with chronic SCI. Our primary objective is to determine a suitable autologous cell source in these patients that is capable of regenerating the vascular bed.

The sample size for group comparisons was determined based on the probability of obtaining a sufficient number of cells per patient for the planned experiments. Previous studies have demonstrated that at least 1 g of adipose tissue enables the isolation of over 80 million functional MSCs/ECFCs in vitro and in vivo in 100% of cases after one month of expansion [28]. Given the high success rate of this approach, a power threshold > 0.90 was assumed to optimize feasibility while maintaining statistical robustness. Under these conditions, a final sample size of 15 subjects per group was determined to ensure reliable results while minimizing the risk of type II errors.

**Fig. 1:**
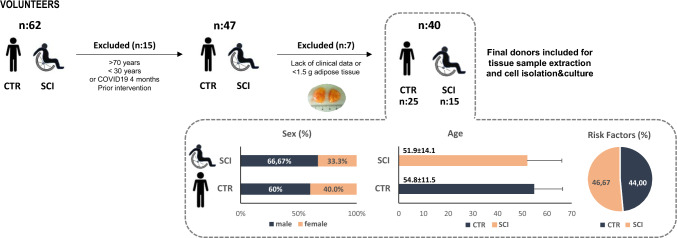
Patients recruitment, characteristics of the study population per group: The figure includes the number of donors recruited at the HNP, as well as the exclusion causes and the final number of donors included for tissue sample extraction. Graphical representation of gender (%), age (mean ± SD) and percentage of patients with associated risk factors (%) is shown below for the 40 donors providing AT samples

Patients with SCI and chronic PIs (grade 3 or 4) were selected, while the control group consisted of individuals without SCI or PIs, matched by age and sex. The control group (CTR) included individuals both with and without known risk factors. From the initial cohort (n = 62), exclusions were made for individuals younger than 30 or older than 70 years, those with a history of COVID-19 within the four months preceding the intervention (n = 15), and those with insufficient data or AT samples yielding less than 1.5 g (n = 7) (Fig. 1). The COVID-19 exclusion criterion was introduced following previously published evidence and our own data indicating that recent SARS-CoV-2 infection can induce transient molecular and cellular alterations in adipose tissue [34]. These changes have been associated with impaired endogenous repair mechanisms and could potentially compromise the regenerative behavior of adipose-derived cell populations such as MSCs and ECFCs.

For the experimental design, we analyzed and compared AT-derived cellular fractions previously identified for their therapeutic potential. The most effective combination for vascular bed formation within an implant was determined. A cross-sectional comparative study was then conducted to evaluate differences between SCI and non-SCI groups. Participants were randomly assigned to experimental conditions, ensuring that only age and sex were matched across groups.

### Extraction of the SVF from human subcutaneous WAT

Samples of subcutaneous WAT were obtained during clinically indicated procedures following approved protocols at each participating hospital center. WAT samples of 1.5 to 5 g were harvested, washed thoroughly, and enzymatically digested using collagenase and dispase for 1.5 h at 37 °C. Post-digestion, WAT samples were subjected to centrifugation at 2500 × *g* for 10 min to separate mature adipocytes, which were discarded in the supernatant. The resulting pellet was resuspended in D10 medium, which consisted of high glucose Dulbecco's Modified Eagle Medium (DMEM, Cytiva) supplemented with 10% fetal bovine serum (FBS, Merck), 1% glutamine and penicillin–streptomycin (GPS, Gibco). Freshly isolated SVF cells were either used immediately for further analyses or stored appropriately for subsequent experimental approaches.

### Isolation of ASCs from AT-SVF

Next, freshly isolated SVF cells were plated at a density of 18,000 cells/cm^2^ on 1% gelatin-coated dishes and cultured at 37 °C in an atmosphere of 5% CO_2_ in humid air, using D10 culture medium. The adherence and proliferation capacity of certain cell populations within the SVF allowed the obtention of more homogeneous populations rich in MSCs, identified as ASCs. Once the adherent ASCs reached confluence (6–8 days), they were detached using TrypLE (Gibco) and split into two portions. In each passage, one fraction was expanded in culture, while the other was cryopreserved. ASCs were expanded over 15 days, with all passages performed by plating cells on 1% gelatin-coated culture plates at a density of 18,000 cells/cm^2^ using D10 medium. The culture medium was refreshed every 3 days, and cells were trypsinized and replated under the same conditions for consecutive passages. Cumulative total cell numbers were determined by hemocytometer cell counting at the end of each passage. In each passage, 1 million cells were cryopreserved in 1 ml of cryopreservation medium (10% DMSO in FBS) and stored in liquid nitrogen until use. This process ensured a steady supply of homogeneous ASCs for further experimental applications.

### Isolation of ECFCs and MSCs from WAT

ECFCs and MSCs were purified from the SVF obtained of 1.5 g of WAT as described above, following a previously published protocol [28], with some modifications. Briefly, ECFCs were purified using magnetic-activated cell sorting (MACS) with CD31-coated magnetic beads (Dynabeads, Invitrogen). The CD31-selected ECFCs were cultured on 1% gelatin-coated plates using ECFC medium: EGM-2 (excluding hydrocortisone; Lonza, Walkersville, MD) supplemented with 20% FBS, 1% GPS. MSCs were obtained from the CD31-negative fraction of the SVF and cultured on coated plates using MSC medium: MSCGM-2 (Promocell), supplemented with 10% FBS and 1% GPS. The described methodology ensures the isolation and purification of specific cell populations from WAT for subsequent analysis and experimentation.

### Expansion potential and cryopreservation

The expansion potential of ECFCs and MSCs was systematically assessed through a common set of passage protocols. For each cell type, cells were seeded on 1% gelatin-coated plates with medium refreshment every 2–3 days. The seeding densities and respective culture media were as follows: ECFCs: 18.000 cells/cm^2^ using ECFC medium over a 32-day expansion period; MSCs: 9.000 cells/cm^2^ using MSC medium over a 28-day expansion period.

Harvesting was accomplished through trypsinization, and subsequent passages involved reseeding the harvested cells under the same conditions. Cumulative cell numbers were determined at the conclusion of each passage using a hemocytometer. In each passage, 1 million cells per ml of cryopreservation medium were cryopreserved and stored in liquid nitrogen until further use.

### Flow cytometry

Flow cytometry analyses were conducted to confirm cell identities, using the following antibodies: APC-conjugated mouse anti-human CD31 (Biolegend), PE-conjugated mouse anti-human CD90 (Biolegend), and FITC-conjugated mouse anti-human CD45 (BD Biosciences) at a dilution of 1:50. The antibody labeling process took place for 20 min in ice, followed by three washes with 1% BSA, 1% FBS, 0.2 mM EDTA in PBS, and resuspension in 1% paraformaldehyde in PBS. Flow cytometric analyses were executed using an Aria flow cytometer (BD Biosciences) and analyzed with FlowJo software. To ensure the specificity of the antibodies, MSCs were used as controls during the characterization of ECFCs, and conversely, ECFCs served as controls during the characterization of MSCs.

### Indirect immunofluorescence

Immunofluorescence was carried out using mouse anti-human CD31 (1:70; Biolegend), mouse anti-human CD73 (1:50; BD Pharmigen), mouse anti-human CD90 (1:70; Biolegend) and mouse anti-human vWF (1:200; Dako) antibodies, followed by Alexa Flour 488 and 555 donkey anti-mouse conjugated secondary antibody (1:1000; Vector) and cell nuclei were counterstained with Hoechst (Invitrogen).

### Endothelial colony-forming assay

To assess the clonogenic potential of endothelial colony-forming cells (ECFCs), passage 4 (P4) cells were seeded at clonal density (5 cells/cm^2^; ~ 300 cells per plate) onto 1% gelatin-coated 100 mm culture dishes (surface area ~ 58 cm^2^), using ECFC-specific medium (n = 3 per group). Medium was replaced every 3 days. After 12 days, colonies containing 25 or more cells were identified by fluorescence microscopy following nuclear staining with DAPI. Endothelial identity was confirmed by staining with Alexa Fluor® 488-conjugated Ulex europaeus agglutinin I (UEA-1; 1:100). Colonies were counted manually under a fluorescence microscope.

### In vitro angiogenesis assays

The formation of vascular networks in vitro was assessed using Matrigel (BD Biosciences). ECFCs were seeded onto Matrigel-coated dishes at a density of 2 × 10^4^ cells/cm^2^ and cultured for 24 h in ECFC medium. Images were captured using a phase-contrast microscope, and the total number of capillaries per well (28 cm^2^) was quantified using ImageJ analysis software (Java).

### Scratch assay

A scratch assay was conducted on fully confluent ECFCs cultures plated in 12-well plates. Scratch wounds were created across each well using a pipette tip. Subsequently, cells were treated for 24 h with basal medium either in the presence or absence of 1 ng/mL of FGF-2 (Gibco). The scratch size was monitored over 4 h under a microscope equipped for Video Time-Lapse imaging.

### E-selectin and VCAM-1 up-regulation after TNF-α exposure

ECFC monolayers were exposed to either 10 ng/mL of tumor necrosis factor-α (TNF-α) or left untreated for 5 h. Next, the analysis of surface antigens was conducted using flow cytometry with antibodies targeting human E-selectin (CD62E-PE) and VCAM-1 (CD106-APC) at a dilution of 1:100 (BD Biosciences).

### Adipogenic differentiation of MSCs

Fully confluent MSCs were cultured over a 10-day period in low-glucose DMEM supplemented with 10% FBS, 1% GPS, and adipogenic supplements (5 μg/mL insulin, 1 μM dexamethasone, 0.5 mM isobutylmethylxanthine, 60 μM indomethacin and 1 μM rosiglitazone). Adipocyte differentiation was assessed through Oil Red O staining. As a negative control, the same cells were cultured in medium without adipogenic supplements.

### Osteogenic differentiation of MSCs

Fully confluent MSCs were cultured for 20 days in low-glucose DMEM supplemented with 10% FBS, 1% GPS, and osteogenic supplements (1 μM dexamethasone, 10 mM β-glycerophosphate, 60 μM ascorbic acid-2-phosphate). Osteogenic differentiation was evaluated through alkaline phosphatase staining. For negative control purposes, the identical cells were cultured in a medium lacking osteogenic supplement.

### Chondrogenic differentiation of MSCs

Suspensions of MSCs were gently centrifuged in 15 mL polypropylene tubes (500,000 cells/tube). The resulting pellets were cultured in high-glucose DMEM medium supplemented with 1% GPS and chondrogenic supplements (1% insulin–transferrin–selenium, 1 μM dexamethasone, 100 μM ascorbic acid-2-phosphate, and 10 ng/mL TGF-β3). After a 20-day culture period, the pellets were fixed in 10% buffered formalin, cryoprotected in a 30% (w/v) sucrose solution, embedded in O.C.T. medium, and sectioned (9 μm-thick) using a cryostat microtome. Chondrogenic differentiation was assessed by examining the presence of glycosaminoglycans following Alcian Blue staining. Negative controls, lacking TGF-β3, did not form compact spheroids.

### In vivo vasculogenic assay

Five-week-old male BALB/c nude (CAnN.Cg-Foxn1^nu^/Crl) mice were sourced from Charles River Laboratories and housed in a dedicated animal facility at the HNP. These facilities were equipped with constant monitoring of air quality, including filters and recirculation, and vigilant technical staff to promptly address any signs of health issues. Mice received a sterile standard diet and had access to sterile water throughout the study. Ethical approval for experimental procedures was obtained from the Ethical Committee for Animal Research (CEEA) at the HNP and the Castilla-La Mancha committee of animal experimentation (JGH. Servicio Ganadería, 142020). The study adhered to standard guidelines for animal research outlined in Spanish laws (RD 53/2013) and European Regulations (2020/569/EU).

In total, three experimental approaches were conducted with different cell combinations (n:5 per condition): (i) 2 × 10^6^ MSCs, (ii) 2 × 10^6^ ASCs, and (iii) 2 × 10^6^ ECFCs and MSCs at a 40:60 ECFCs/MSCs ratio. Each cell mixture was resuspended in 200 μL of ice-cold Phenol Red-free Matrigel (BD Biosciences), and subcutaneously injected into the dorsal region of the 6-weeks mice.

### Histology and immunohistochemistry

Seven days after the subcutaneous injection, implants were retrieved from euthanized mice, fixed in 10% buffered formalin overnight, processed for paraffin embedding, and cut into 5-μm-thick sections. Hematoxylin and eosin (H&E) staining was performed to evaluate the presence of blood vessels containing red blood cells. For immunohistochemistry, sections underwent deparaffinization, and antigen retrieval was performed by heating the sections in Tris–EDTA buffer (10 mM Tris-Base, 2 mM EDTA, 0.05% Tween-20, pH 9.0). These sections were blocked for 30 min in 5–10% blocking serum and then incubated over night at room temperature with the primary antibodies: mouse anti-human CD31 (1:400; GeneTex) and mouse anti-human α-SMA (1:200; Sigma-Aldrich, A2547 Clone 1A4). Detection of hCD31 involved the use of horseradish peroxidase (HRP)-conjugated mouse secondary antibody (1:1000; Vector Laboratories) and 3,3′-diaminobenzidine (DAB), followed by hematoxylin counterstaining and Permount mounting. Fluorescent staining was performed using FITC-conjugated UEA-1 (20 μg/mL) and FITC-conjugated secondary antibodies (1:200; Vector Laboratories), followed by Hoechst counterstaining. Image processing was carried out using ImageJ (NIH, USA) to remove autofluorescence captured in the 633 nm channel, which was acquired without any antibody staining and used solely to detect endogenous autofluorescence (e.g., from red blood cells). This signal was subtracted from the nuclei, CD31, α-SMA and UEA-1 channels using the “Image Calculator” function. The resulting images were then recombined and adjusted for brightness and contrast. All images from both SCI and CTR groups were processed under identical conditions.

### In vivo quantification of human vascular cells

To perform a quantitative analysis, some of the implants were extracted after 7 days and enzymatically digested with collagenase and dispase for 1 h at 37 °C. Retrieved cells were analyzed by fluorescence-activated cell sorting (FACS) following incubation with FITC-conjugated anti-mouse CD45, APC-conjugated anti-human CD90, and PE-conjugated anti-human CD31 antibodies (all 1:50; BD Biosciences). ECFCs were identified as mCD45-/hCD31 + /hCD90−, while MSCs were classified as mCD45−/hCD31−/hCD90 +.

### Statistical analysis

The results are presented as mean ± SD. Pairwise comparisons were conducted using Student’s t-tests, performed with GraphPad Prism software (Version 8). A *p*-value < 0.05 was considered statistically significant. For multiple comparisons, appropriate corrections were applied to control for type I error.

## Results

### Heterogeneity and low survival of SVF cells

**Fig. 2:**
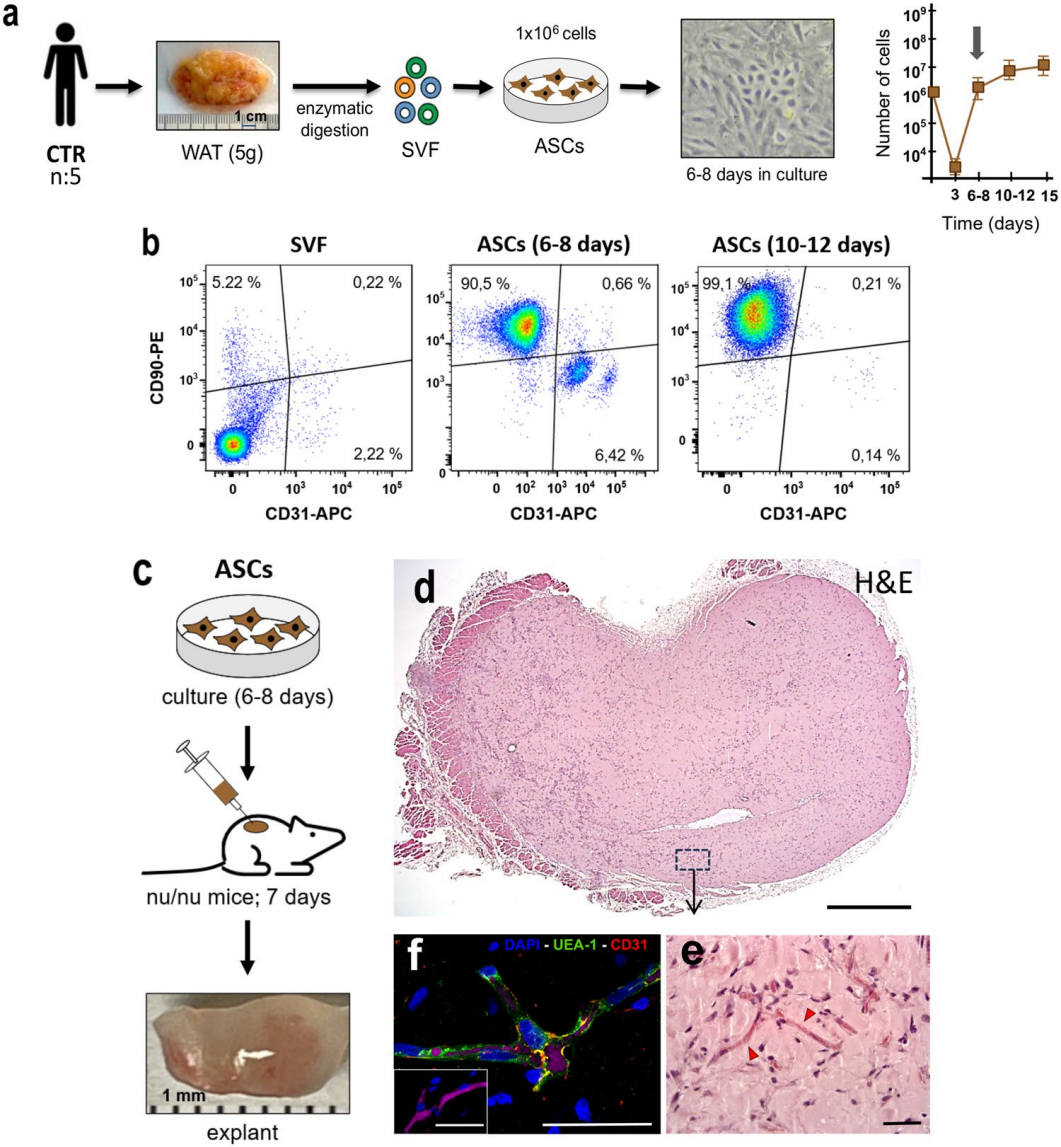
Characterization of the SVF and ASCs cell fractions: **a** Samples were obtained from 5 control subjects, and adipose tissue was processed to isolate the stromal vascular fraction (SVF) via enzymatic digestion. The SVF was then cultured, and adherent stromal cells were expanded. By days 6–8 of culture, a confluent monolayer revealed two distinct cell populations: one with cobblestone-like morphology characteristic of ECFCs, and another with spindle-shaped morphology characteristic of MSCs. The cells were cultured and expanded for a total of 15 days, as shown in the graph. **b** Flow cytometry analysis revealed that the SVF contained less than 10% CD90⁺/CD31⁺ cells, while ASCs analyzed after 6–8 days of culture showed a heterogeneous population predominantly enriched in CD90⁺ cells, with a smaller proportion of CD31⁺ cells. By days 10–12, the ASC population became almost entirely CD90⁺, reaching up to 99.1%. **c** ASCs cultured for 6–8 days were injected subcutaneously into nude mice and explanted after 7 days. The extracted implants displayed a reddish surface appearance. (**d**–**e**) Hematoxylin and eosin (H&E) staining: **d** complete explant sectioned transversely; **e** representative magnification showing numerous blood vessels (red arrowheads), predominantly located at the periphery of the implant. **f** Immunofluorescence confirmed the presence of human endothelial structures via co-staining with UEA-1 (green) and anti-human CD31 (red), indicating the human origin of the endothelial lining. A negative control for human CD31 is shown in the inset. Intraluminal red blood cells appear magenta due to their natural autofluorescence. Scale bars: 1 mm (**d**), 0.2 mm (**e**), 50 μm (**f**)

SVF cells obtained directly from WAT enzymatic digestion of control subjects (Fig. 2a) were analyzed by flow cytometry, revealing a highly heterogeneous cell population (Fig. 2b). Fewer than 10% of the SVF cells expressed CD31 (endothelial marker), CD90 (mesenchymal marker), or CD45 (hematopoietic marker) (Fig. S1a). Moreover, the composition of SVF cells, based on the expression of CD31, CD90, and CD45, showed variability across the five samples analyzed (Fig. S1a). Additionally, the post-cryopreservation survival rate of these cells was consistently low (< 30%) in all five samples (Fig. S2).

### Isolation and characterization of ASCs from WAT

ASCs derived after SVF seeding in 1% gelatin-coated plates exhibited a heterogeneous morphology, primarily composed of two cell populations: spindle-shaped cells characteristic of MSCs and colony-forming cells with cobblestone-like morphology characteristic of ECFCs (Fig. 2a).

To evaluate the expansion potential of ASCs, these cells were subcultured for 15 days. Starting with 1 million SVF cells, approximately 10 million ASCs were obtained within 15 days, although a reduced proliferation rate was observed starting from days 6–7 of culture (Fig. 2a). Twenty-four hours after seeding, over 90% of the cells remained in suspension and were removed during the first medium change at 72 h (3 days). The adherent cells exhibited clonal ability, reaching confluence within 6–8 days of culture. These adherent cells, now identified as ASCs, revealed a population enriched in CD90 and CD31 during the early days (6–8). Flow cytometry analysis confirmed the immunophenotypic profile of ASCs based on CD90, CD31 and CD45 expression across five independent samples (Fig. S1b). However, upon subculturing the same cells to continue expansion, by day 10 of culture 99% of the cells displayed the CD90 + /CD31 − /CD45 − phenotype (MSCs) (Fig. 2b). Notably, unlike SVF cells, whose cryopreservation resulted in the loss of over 70% of the population, more than 90% of ASCs survived this process (Fig. S2).

### Evaluation of the vasculogenic potential of purified ASCs

The vasculogenic potential of ASCs was also evaluated in an in vivo approach in which 2 × 10^6^ ASCs were subcutaneously implanted in nude mice (Fig. 2c). Seven days after implantation, histological analysis revealed the formation of perfused blood vessels (Fig. 2d, e) predominantly at the periphery of the implant in 2 out of 5 implants analyzed (Fig. S3a). Immunohistochemical staining with human CD31 and UEA-1 further confirmed the presence of vessels lined with human ECFCs (Fig. 2f).

### Isolation and characterization of ECFCs and MSCs from WAT

**Fig. 3:**
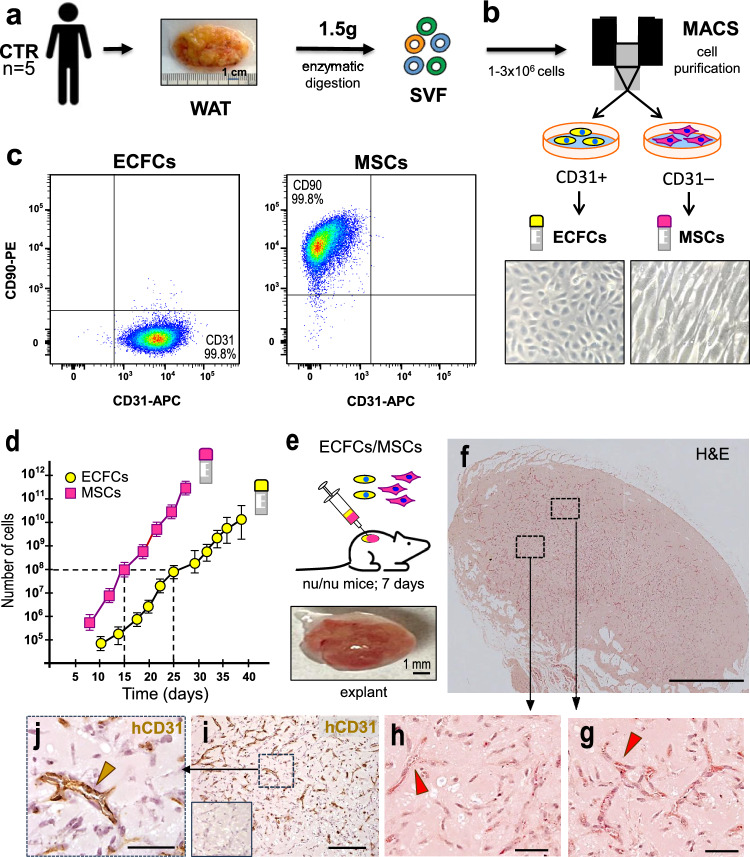
Characterization of the ECFC/MSC combination: **a**, **b** The stromal vascular fraction (SVF) from adipose tissue of 5 control subjects was obtained by enzymatic digestion. Cells were separated using anti-CD31 magnetic beads, yielding two distinct populations: **a** CD31⁺ fraction corresponding to ECFCs and a CD31^−^ fraction corresponding to MSCs. **c** Flow cytometry analysis confirmed high purity of both populations, with more than 99% of ECFCs and MSCs expressing CD31 and CD90, respectively. **d** The expansion capacity of each purified population was evaluated. Both cell types reached approximately 10^8^ cells, with ECFCs doing so in 25 days and MSCs in 15 days. **e** The vasculogenic potential of the ECFC/MSC combination was assessed in vivo by subcutaneous implantation into nude mice. **f** Hematoxylin and eosin (H&E) staining of a complete transverse section of the implant revealed widespread vascular structures throughout the tissue. **g**–**h** Representative magnified views of H&E-stained sections show numerous perfused blood vessels (red arrowheads). **i**–**j** Immunohistochemical staining using anti-human CD31, followed by HRP-conjugated secondary antibody and DAB chromogen (brown), confirmed the presence of human endothelial cells forming capillaries (brown arrowheads). A negative control for human CD31 is shown in the inset. Scale bars: 1 mm (f), 0.2 mm (**i**), 50 µm (**g**–**j**)

The same WAT from control subjects (n = 5) used for the isolation of the SVF and ASCs, was used to isolate and amplify the resident ECFCs/MSCs and determine their vasculogenic potential (Fig. 3a). The purification of ECFCs from the SVF using CD31-coated magnetic beads (Fig. 3b) allowed us to separate two cell populations through subsequent routine cultures, corresponding to the CD31 + (ECFCs) and CD31- (MSCs) fractions. In both cases, cells were grown separately until reaching confluence, obtaining two homogeneous populations that displayed morphological characteristics consistent with their respective cellular identities (Fig. 3b). Passage 0 (P0) was established for ECFCs when they showed uniform expression of CD31 + (higher than 96%), with an insignificant presence of CD90 + MSCs or CD45 + hematopoietic cells (fewer than 1% in both cases). For MSCs, P0 was defined when the uniform expression of CD90 exceeded 96% (CD90 + /CD31−/CD45−) (Fig. 3c). ECFCs were serially expanded up to passage 11, and MSCs up to passage 7, to confirm that sufficient cell numbers could be reached within a clinically relevant time frame. We obtained approximately 100 million cells for each type: ECFCs in about 25 days and MSCs in 15 days (Fig. 3d). To ensure the identity of purified cells, additional phenotypic and functional tests were conducted for each purified population. As in previous studies [28], this methodology proved to be reproducible, resulting in the successful isolation of ECFCs and MSCs in all processed WAT samples (5/5). Moreover, post-cryopreservation survival exceeded 95% across all analyzed samples (Fig. S2).

### Assessment of the vasculogenic potential of purified MSCs

The vasculogenic potential of isolated MSCs was also evaluated in vivo in matrigel implants as described above. Seven days post-implantation, histological analysis revealed the absence of blood vessels within the interior of the implants (Fig. S3b). Indeed, blood vessels were detected in only 1 out of 5 implants, localized at the most peripheral region of the implant.

### Combined vasculogenic potential of purified ECFCs and MSCs

Finally, ECFCs were combined with MSCs (at a 40:60 ratio) and subcutaneously implanted in nude mice for 7 days (Fig. 3e), as previously described [28]. Histological examination revealed the presence of networks of perfused microvessels throughout all the implants (Fig. 3f–h and S3c), with the localization of human ECFCs confirmed through specific hCD31 immunostaining (Fig. 3i, j). In contrast to previous approaches, in this case, revascularization was observed in all 5 implants studied (Fig. S3c).

### Vasculogenic potential of purified ECFCs/MSCs from chronic SCI patients WAT

**Fig. 4:**
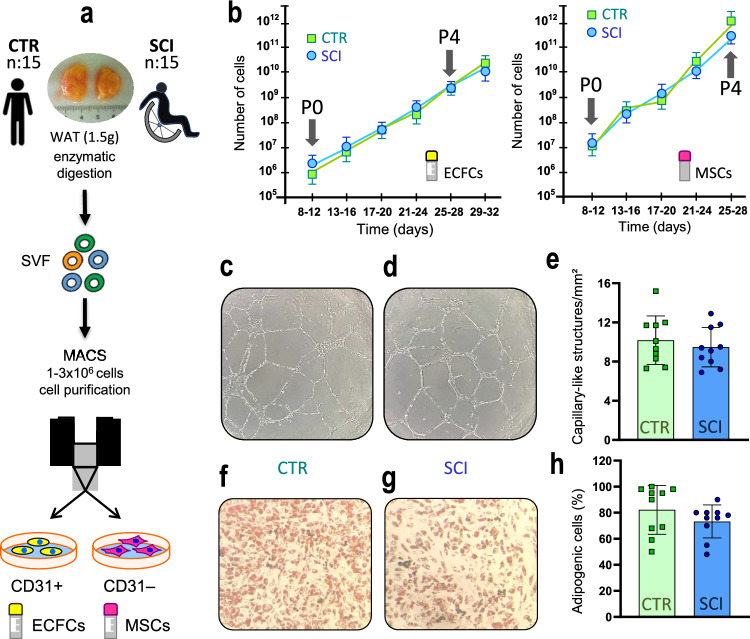
Comparison of ECFCs and MSCs isolated from SCI patients or non-SCI controls: **a** Adipose tissue (1.5 g) from control subjects (n = 15) and spinal cord injury (SCI) patients (n = 15) was used to obtain the stromal vascular fraction (SVF) and to purify ECFCs and MSCs. **b** No significant differences were observed in the expansion capacity of ECFCs and MSCs between the control and SCI groups. **c**-**e** Representative micrograph of an ECFC-lined capillary-like network on a Matrigel-coated plate, with total capillaries quantified after 12 h, assessing vasculogenic capacity. **f**–**h** Similarly, a representative micrograph shows MSCs differentiating into adipocytes on a culture plate, with the percentage of preadipocytes covering the plate quantified after 10 days of differentiation. **c**–**h** Although an apparent decrease in both vasculogenic capacity and adipogenic differentiation was observed in SCI subjects compared to controls, no statistical differences were detected

Lastly, we evaluated the vasculogenic potential of ECFCs and MSCs purified from WAT isolated from SCI patients, Fig. 1 shows anthropometric and risk variables in the studied population, highlighting no significant differences in sex or age between SCI or control individuals. To enhance group comparability, subpopulations of ECFCs and MSCs were extracted from an equivalent amount of WAT per patient (Fig. 4a).

Both cell populations exhibited comparable expansion potential in SCI patients and controls, without significant differences in yield (Fig. 4b). The phenotypic characteristics of ECFCs and MSCs were consistent across both groups, showing similar marker expression profile: CD45 −/CD31 + /CD90 − for ECFCs (Fig. S3a) and CD45 −/CD31 −/CD90 + for MSCs (Fig. S4a). ECFCs from both control and SCI patients displayed comparable morphology, expressed CD31 and von Willebrand factor (vWF) (Fig. S4b), and maintained their clonal growth ability by forming colonies from single cells, a hallmark feature of endothelial progenitors not observed in mature endothelial cells (Fig. S4c). Similarly, MSCs expressed CD90 and CD73 across groups (Fig. S5b). Functionally, both ECFCs and MSCs retained key capacities in vitro. ECFCs from both groups exhibited similar migratory potential (Fig. S4d) and responded to TNF alpha stimulation by upregulating VCAM 1 and E selectin (Fig. S4e). MSCs retained their ability to differentiate into osteogenic, chondrogenic, and adipogenic lineages (Fig. S5c).

Although a consistent trend toward slightly lower values was observed across multiple functional assays in the SCI group, none of these differences reached statistical significance. These findings suggest that, within the resolution of our current analyses, SCI derived cells retain functional capacities comparable to those of control cells (Fig. 4e, h).

Finally, the in vivo vasculogenic potential of ECFCs and MSCs derived from CTR and SCI patients was evaluated using seven donor-matched implants per group, generated by co-injecting both cell types embedded in Matrigel into immunodeficient mice. Two complementary approaches were used for evaluation: histological analysis and enzymatic digestion followed by flow cytometry.

**Fig. 5:**
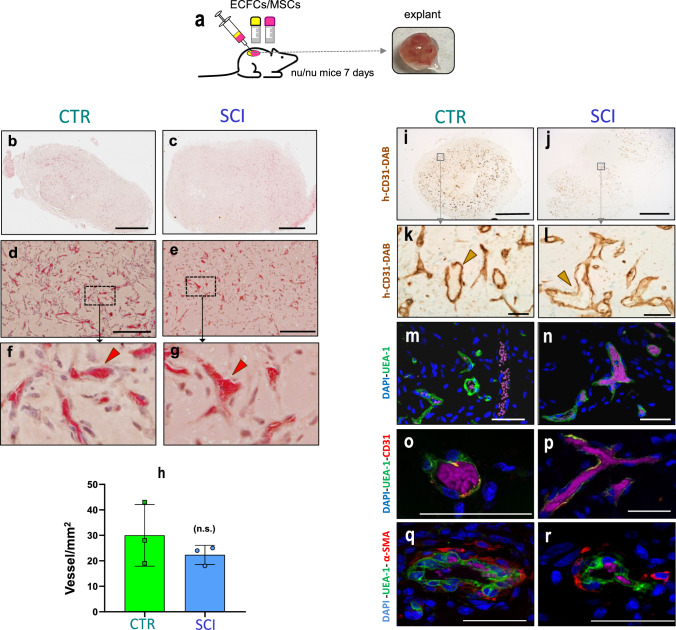
In vivo vasculogenic potential of ECFCs/MSCs: **a** Schematic illustration of experimental approach: implants were fixed for histological analysis 7 days post-transplantation. **b**, **c** Low-magnification H&E-stained transverse sections of entire implants from representative CTR and SCI samples, showing widespread vessel formation. **d**, **e** Intermediate magnification images highlighting blood vessel presence within the implants. **f**, **g** High-magnification images showing perfused microvessels containing intraluminal red blood cells (red arrowheads). **h**) Quantification of vessel density based on the number of erythrocyte-containing lumens per mm^2^ in three implants per group. No statistically significant differences were observed between groups. **i**–**l** Immunohistochemistry using anti-human CD31 antibodies reveals DAB-positive luminal structures (brown arrowheads), confirming the presence of human endothelial cells forming vessels. **m**–**n** Immunofluorescence staining for UEA-1 (green), a marker specific to human endothelium. In panel (**m)**, two adjacent vessels are observed: one UEA-1⁺ containing intraluminal red blood cells (RBCs, visible in magenta due to their autofluorescence), and one UEA-1^−^, consistent with a murine origin. **o**–**p** Double immunofluorescence for UEA-1 (green) and human CD31 (red) confirms co-localization in human-derived vascular structures. **q**, **r** Perivascular coverage is evidenced by co-staining with UEA-1 (green) and α-SMA (red), indicating the presence of smooth muscle cells around human vessels. Cell nuclei are counterstained with DAPI (blue). Scale bars: 1 mm (**a**, **b**, **i** and **j**), 0.2 mm (**d**–**e**), and 50 μm (**k**–**r**)

Macroscopically, the implants exhibited a reddish appearance, indicative of blood perfusion (Fig. 5a). Histological analysis revealed extensive networks of perfused microvessels in both groups (Fig. 5b–g), with the presence of intraluminal red blood cells confirming vessel functionality. Quantification of vessel density showed no statistically significant differences between groups (Fig. 5h).

The presence of human endothelial cells forming vascular structures was confirmed by immunohistochemistry for human CD31 and by UEA-1 staining, which allowed discrimination of human-derived vessels from murine ones (Fig. 5i–p). Co-staining with α-SMA indicated perivascular coverage, supporting the formation of more mature and stabilized vessels in both groups (Fig. 5q–r).

**Fig. 6:**
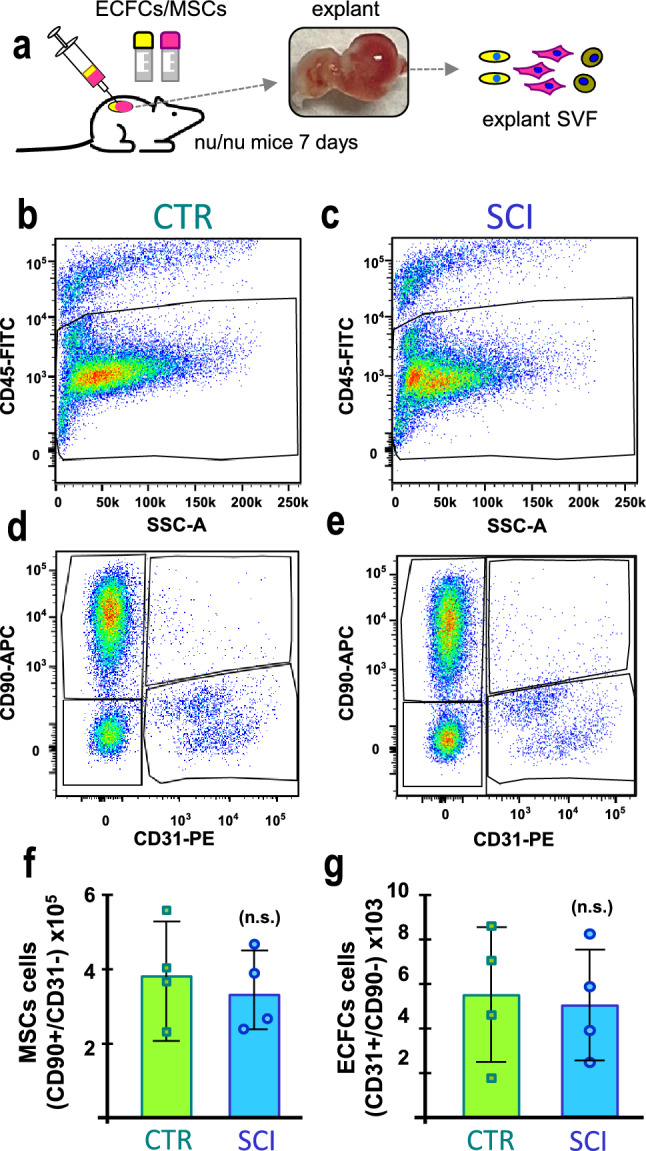
Quantification of human ECFC and MSC integration into implants. **a** Schematic representation of the experimental approach: ECFCs and MSCs from CTR and SCI groups were injected subcutaneously in Matrigel into nude mice. After 7 days, the implants were enzymatically digested to isolate the stromal vascular fraction (SVF), which was subsequently analyzed by flow cytometry. **b**, **c** Representative FACS plots showing CD45 versus SSC-A from digested implants of each group (CTR and SCI), used to exclude hematopoietic cells. **d**, **e** Gating strategy applied to the CD45^−^ population to identify human MSCs (CD90⁺/CD31^−^) and ECFCs (CD31⁺/CD90^−^), using CD90-APC and CD31-PE markers. **f**, **g** Quantification of human cells integrated into the implant. Bar graphs show the average number of MSCs (CD90⁺/CD31^−^, left; × 10^5^ cells) and ECFCs (CD31⁺/CD90^−^, right; × 10^3^ cells) per group. No significant differences were found between CTR and SCI groups

To complement these findings with a quantitative assessment of cell integration, four additional implants per group were enzymatically digested and analyzed by flow cytometry (Fig. 6a). Human ECFCs and MSCs were identified within the CD45^−^ population as CD31⁺/CD90^−^ and CD90⁺/CD31^−^ cells, respectively (Fig. 6b–e). No significant differences were observed in the number of human cells recovered per implant between groups (Fig. 6f–g).

## Discussion

In recent decades, the field of regenerative medicine has undergone significant progress, with a prominent role being assumed by adipose-derived cells in advanced therapies, emerging as a central strategy for the regeneration of damaged tissues [35–37]. Significant scientific and clinical attention has focused on the use of SVF from AT [38], as well as on the resident cells within this fraction, such as MSCs [39] and ECFCs [28], which are capable of regenerating vascular beds and adjacent tissues [26, 40]. However, despite the remarkable therapeutic potential demonstrated by these cells, numerous studies indicate that various pathologies can significantly influence their behavior and function [24, 41–43]. In this study, we confirmed that the purification and separated expansion of ECFCs and MSCs extracted from SVF enhances their efficacy when they are afterwards transplanted together to promote functional vascular beds in vivo, compared to the use of ASCs obtained from the direct expansion of the same SVF. Furthermore, we have shown that, in agreement with similar results with cells from control subjects [28], MSCs and ECFCs purified from the SVF of subcutaneous WAT of patients with SCI and PIs are functional both in vitro and in vivo in the context of vascular morphogenesis.

### Therapeutic potential of resident cells in AT

As stated above, AT-derived SVF have become one of the most widely used cell sources in clinical practice due to its easy accessibility with low morbidity and its rich cellular composition, which includes adipocytes, stem cells, hematopoietic cells, and ECs, among others [36, 38, 44–47]. These characteristics make AT a highly valuable resource for promoting angiogenesis, increasing vascular density, and improving blood supply in ischemic tissues [47, 48].

The AT-derived SVF is also richer in colony-forming cells, including MSCs, compared to the SVF obtained from bone marrow [49]. However, our data consistent with previous studies [49, 50], show that fewer than 10% of the cells are positive for CD90/CD31 markers which are characteristic of MSCs and ECFCs, respectively [28]. Additionally, we observed significant variability in the proportions of identified cell populations (CD90 + CD31 − CD45 − or CD31 + CD90 − CD45 −) across the samples analyzed, highlighting the intrinsic heterogeneity of these cellular populations [51]. Further compounding these challenges is the low cellular viability after cryopreservation, with mortality rates exceeding 70% across all samples during the freezing–thawing process. This combination of cellular heterogeneity, sample variability, and low survival rates after cryopreservation undermines the functional analysis of the SVF and its reproducibility, making comparative studies between patients particularly difficult.

Therefore even though the analysis involved only five samples, we find that the results obtained by the direct use of the SVF are unpredictable and not consistent, making it challenging to anticipate its therapeutic efficacy across different individuals.

Alternatively, we evaluated the potential use of ASCs directly obtained after culturing the SVF in vitro, a population phenotypically characterized by markers such as CD13, CD73, CD90, CD105, CD31, CD45, and CD235a [50]. This heterogeneous population of adherent cells, primarily composed of MSCs, has attracted significant interest in regenerative medicine due to its potential to promote angiogenesis and vasculogenesis. Our study revealed that cultured ASCs predominantly contain two cellular populations, identified as MSCs and ECs with MSCs comprising 90% of the cells and ECs representing 3–7%. These findings are consistent with previous reports [50] and underscore the MSCs enrichment of adherent ASCs populations. However, their variable and heterogeneous composition across individuals poses an additional challenge in comparative studies of distinct cell populations.

In vivo, ASCs promoted the formation of perfused vessels in matrigel implants seven days after transplantation into immunocompromised mice. However, this effect was observed only in a subset of implants, and the functional blood vessels were mainly restricted to the peripheral regions of the implants. This pattern is consistent with previous studies using purified MSCs populations [26]. Given that ASCs populations are typically composed of over 90% MSCs, this outcome may be explained by the known paracrine activity of MSCs, particularly their secretion of proangiogenic factors such as VEGF and PDGF [26]. These factors can stimulate the sprouting and ingrowth of host-derived vasculature from surrounding tissues [21, 52]. Nevertheless, such host-driven angiogenesis appears insufficient to support the formation of de novo human vascular networks within the implant core, which instead requires the presence of vasculogenic cells such as ECFCs [26].

In this context, our findings differ from previous studies using purified MSCs alone [26], in a crucial way: we detected newly formed vessels composed of human ECs in some ASC implants, suggesting the occurrence of de novo vasculogenesis. This observation underscores a distinctive potential of ASCs compared to MSCs alone. Unlike MSCs, ASCs are a heterogeneous population that includes ECs in variable proportions. A possible explanation for the variability in vascular outcomes is the inter-individual variability observed in ECs representation within ASC populations, which ranged from 3 to 7% after 8 to 12 days of culture. This variability likely impacts the efficacy of ASCs in forming new vessels. It is possible that implants derived from ASC populations with minimal endothelial representation correspond to those lacking blood vessel formation, as suggested by results observed in previous experiments with different combinations of percentages of ECFCs and MSCs [26, 53]. Conversely, implants with higher percentages of ECs were more likely to succeed in vasculogenesis.

However, this reasoning is inherently limited by the inability to quantify the exact composition of ASCs prior to their injection into the implants. Without a doubt, this is a limitation in the approach used, as we could not validate whether a higher percentage of ECs in the ASC samples correlated with the formation of human vessels in the implant. This limitation stems from the low number of cells obtained using our methodology for purifying ASCs. Prolonged expansion of ASCs in culture generates a pure MSC population, which would compromise the heterogeneity of ASCs. To avoid this, we relied on ASCs cells collected from the first confluent plate obtained after 6–8 days of culture, although this strategy limited the total number of ASCs available for analysis, forcing us to use the entire sample to achieve the 2 × 10^6^ ASCs required per implant for each patient. While extracting larger amounts of adipose tissue could have addressed this limitation, the safety and ethical considerations for the patients did not allow us to follow this approach.

Even so, the heterogeneity of ASCs remains both an opportunity and a challenge. While their composite nature enables them to support diverse regenerative processes, it also introduces variability that can affect therapeutic outcomes. Our findings suggest that optimizing the endothelial component within this combination could enhance their efficacy in vascularization.

Overall, our study demonstrates that the purification, individual expansion, and further use of a combination of ECFCs/MSCs provide a significantly advantageous alternative for the vascularization or revascularization of a subcutaneous implant. Compared to the SVF, the preservation of the isolated cells is viable, with a mortality rate below 5% after cryopreservation. Furthermore, the purification of each cell population is highly stable, achieving a 100% success rate in all cases. Also, the individual expansion of ECFCs/MSCs from an equivalent amount of WAT results in more than 100 million cells from both populations in fewer than 25 days, with extremely pure cultures (> 98%). In the SVF fraction, however, more than 90% of the cells could not be identified using the putative markers for MSCs (e.g., CD90) and ECFCs (e.g., CD31) [28], and the continued culture of the SVF cells for 15 days leads to more than 95% of cells with the CD90 + CD31-CD45- phenotype, with a low expansion potential. Regarding ASCs, although the CD90/CD31 proportions were more homogeneous, we found high variability across samples, which complicated result comparisons and limited our analysis. The ability to obtain an enriched percentage of both cell types from a small tissue sample could ensure autologous and allogeneic therapeutic approaches in a greater number of pathologies and patients.

Finally, we have demonstrated that the use of the ECFCs/MSCs combination compared to ASCs shows greater efficacy in blood vessel formation in vivo, positioning it as the strategy with the greatest vasculogenic potential. The effectiveness of MSC + ECFC transplantation seems primarily based on reciprocal interactions between these two cell populations, which improve initial cell survival and long-term vascular stability. Initially, ECFCs play a critical role in enhancing MSC survival immediately after transplantation [26], by secreting several factors such as PDGF-BB, which significantly improves MSCs viability during this early and vulnerable phase. Increased MSC survival subsequently contributes to an enhanced regenerative potential, including improved adipose tissue formation. Conversely, MSCs provide crucial structural and functional support to ECFCs. They occupy perivascular positions resembling pericytes, stabilizing nascent ECFC-formed vessels and enhancing their durability [54]. On the other hand, MSCs contribute to the robustness and durability of the vascular structures by transferring mitochondria to ECFCs through tunneling nanotubes, thereby enhancing their metabolic resilience and engraftment during the early hypoxic and inflammatory environment of transplantation [40]. Moreover, MSCs can recruit critical host-derived cells such as non-inflammatory neutrophils, necessary for successful integration and anastomosis with the host vasculature, as demonstrated previously [55]. Lastly, the specific MSC to ECFC ratio (60:40), initially identified by Melero-Martin et al. [53], likely enhances these cooperative mechanisms by optimizing spatial proximity and cell-to-cell contact. This ratio ensures efficient reciprocal signaling, stabilizing newly formed vessels and allowing them to mature into robust, functional vascular networks.

### ECFCs/MSCs in patients with SCI

Herein, we demonstrate for the first time that ECFCs and MSCs can be reliably isolated and expanded from a minimal amount of subcutaneous adipose tissue in patients with chronic SCI and advanced pressure injuries (grade 3 or 4). Importantly, these cell populations retain key phenotypic and functional properties comparable to those of age- and sex-matched controls, including clonogenicity, multilineage differentiation potential, and responsiveness to inflammatory stimuli. When combined and implanted in vivo, ECFCs and MSCs derived from SCI patients were capable of forming functional, perfused human vascular networks within seven days, indicating preserved vasculogenic capacity. This finding is particularly relevant from a translational perspective, as SCI patients typically exhibit impaired healing and limited regenerative responses [6]. Our results provide a strong proof-of-concept for the potential use of a patient’s own adipose-derived ECFCs and MSCs in autologous regenerative therapies, particularly in the context of chronic wounds and ischemic complications, where rapid and robust vascularization is critical for clinical success.

## Conclusion

In conclusion, our study has delved into the therapeutic potential of AT-derived cells, particularly ECFCs and MSCs, for the vascularization of implants. We have demonstrated that the combination of ECFCs and MSCs, purified and expanded separately, is more effective in forming a functional vascular bed in vivo than the direct use of ASCs purified from the same AT. Moreover, these cells purified and expanded from patients with SCI and PIs exhibit functionality comparable to cells obtained from age- and sex-matched individuals without SCI or PIs, both in vitro and in vivo. While the SVF is widely accessible, it presents a variable cell composition and a low success rate in cryopreservation, complicating its analysis and clinical application. Culturing the SVF results in a heterogeneous ASC population, mainly composed of MSCs (> 90%), but with low expansion potential and limited efficacy in vascular morphogenesis. In contrast, the separately purification and expansion of ECFCs and MSCs from the same SVF provide homogeneous cell populations with high expansion potential, facilitating the application of autologous therapies in more patients. The subsequent combination of ECFCs and MSCs demonstrates greater efficacy in blood vessel formation in vivo compared to ASCs, highlighting its therapeutic potential in vascular regeneration in clinical settings, especially in patients with chronic SCI and PIs. In summary, our results support the continued research and development of therapies based on the combination of purified ECFCs and MSCs, aiming to improve clinical outcomes in tissue regeneration and the vascularization of subcutaneous implants in patients with various pathologies.

## Supplementary Information

Below is the link to the electronic supplementary material.

Supplementary file1 (PDF 800 KB)

## Data Availability

No datasets were generated or analysed during the current study.
