# Supplementary material for: Vasculogenic potential of adipose tissue derived stem cells from patients with chronic spinal cord injury and pressure injuries
Source: Angiogenesis. 2025 Sep 10;28(4):48. doi: 10.1007/s10456-025-10002-y (PMC12423250; doi:10.1007/s10456-025-10002-y)
Supplement: Supplementary file 1 — Supplementary file1 (PDF 800 KB) [file 10456_2025_10002_MOESM1_ESM.pdf]

Title:

VASCULOGENIC POTENTIAL OF ADIPOSE TISSUE DERIVED STEM CELLS FROM PATIENTS WITH CHRONIC SPINAL CORD INJURY AND PRESSURE INJURIES

Authors: Ángela Santos-De-La-Mata[1,2], Pedro F. Esteban[3,4], Mario Martínez-Torija[1,2], Beatriz Paniagua-Torija[2], Fª. Javier Espino[2,5], Lucía Beltran-Camacho[6], Celia Camacho-Toledano[1,2], Monica Alcobendas-Maestro[7], Fernando Garcia-Garcia[8], Eduardo Molina-Holgado[3,4], Mª Carmen Duran-Ruiz[6,9], Juan M. Melero-Martin[10,11,12], Rafael Moreno-Luna[1,2].

Affiliations:

[1] Pathophysiology and Regenerative Medicine Group, Hospital Nacional de Parapléjicos, SESCAM, 45071 Toledo, Spain.

[2] Pathophysiology and Regenerative Medicine, Instituto de Investigación Sanitaria de Castilla-La Mancha (IDISCAM), Spain

[3] Grupo de Neuroinflamación, Hospital Nacional de Parapléjicos, SESCAM; Toledo, Spain.

[4] Grupo de Neuroinflamación, Instituto de Investigación Sanitaria de Castilla-La Mancha (IDISCAM), Spain; eduardom@sescam.jccm.es.

[5] Plastic and Reconstructive Surgery Service, Hospital Nacional de Parapléjicos. SESCAM; Toledo, Spain.

[6] Biomedicine, Biotechnology and Public Health Department, Cádiz University; 11002 Cádiz, Spain.

[7] Department of Physical Rehabilitation, Hospital Nacional de Paraplejicos, SESCAM; 45071 Toledo, Spain.

[8] Radiodiagnostic Service, Hospital Nacional de Parapléjicos, SESCAM; Toledo, Spain.

[9] Biomedical Research and Innovation Institute of Cadiz (INiBICA); Cadiz, Spain.

[10] Department of Cardiac Surgery, Boston Children's Hospital; Boston, MA 02115, USA.

[11] Department of Surgery, Harvard Medical School; Boston, MA 02115, USA.

[12] Harvard Stem Cell Institute; Cambridge, MA 02138, USA.

Supplementary Materials

This file includes:

Figs. S1 to S5

Flow cytometry profile of SVF and ASCs

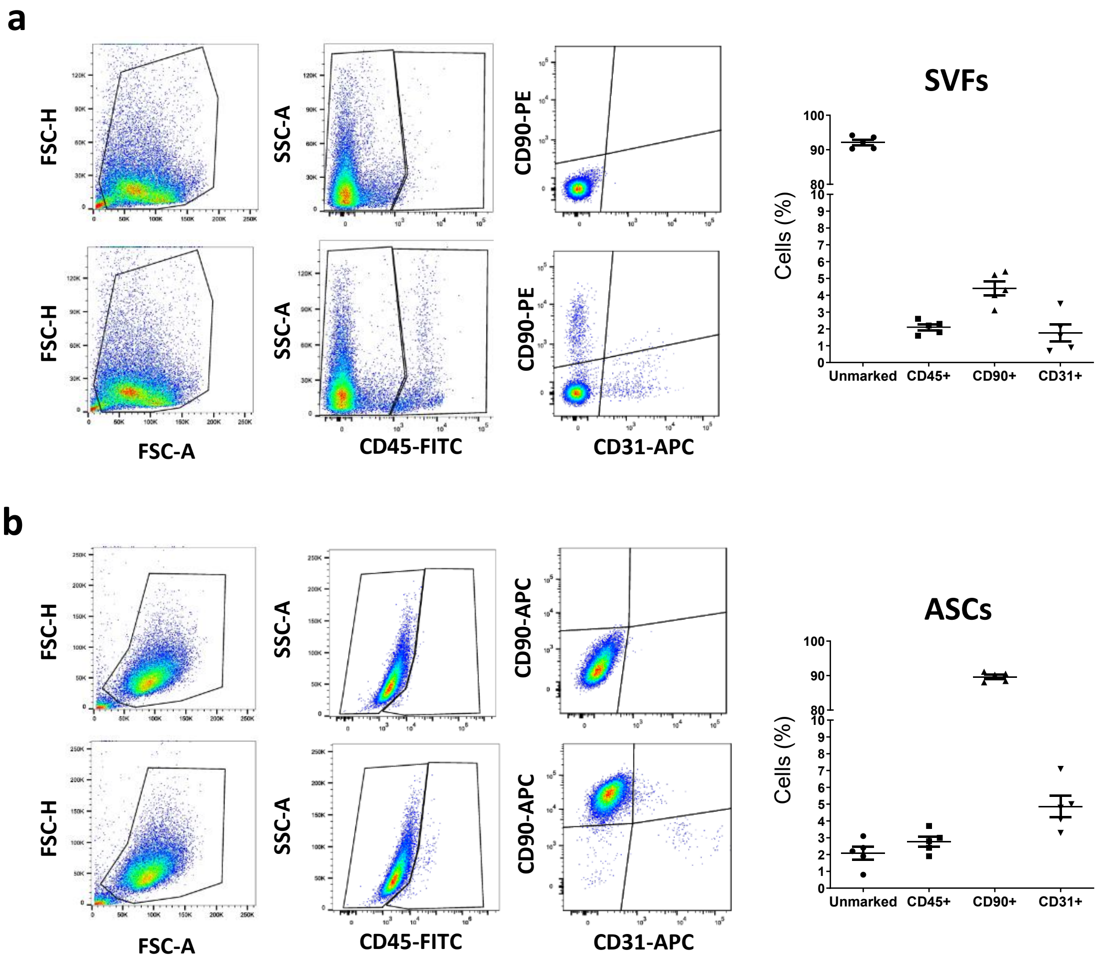

Supplementary Figure S1: Representative dot plots show forward and side scatter (FSC, SSC) gating and surface marker expression for CD45, CD31, and CD90. For each cell type (SVF and ASCs), the upper row displays unstained controls used to define gating parameters, and the lower row shows stained samples. Positive expression was determined based on fluorescence intensity relative to the corresponding unstained control. The accompanying scatter dot plots display the percentage of cells expressing CD45, CD31, or CD90 in each patient sample, with mean values indicated. Cells not expressing any of these markers were classified as “unlabeled. a) SVFs; b) ASCs.

Post-Cryopreservation Cell Viability Assessment

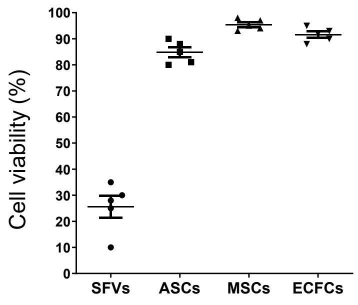

Supplementary Figure S2: Cell Viability. The graphs depict the percentage of viable cells after cryopreservation for SVF, ASCs, MSCs, and ECFCs. For viability assessment, 1×10^6 cells/mL were cryopreserved. After thawing, cells were stained with trypan blue and counted using a Neubauer chamber. Only non-stained (viable) cells were included in the survival percentage calculation.

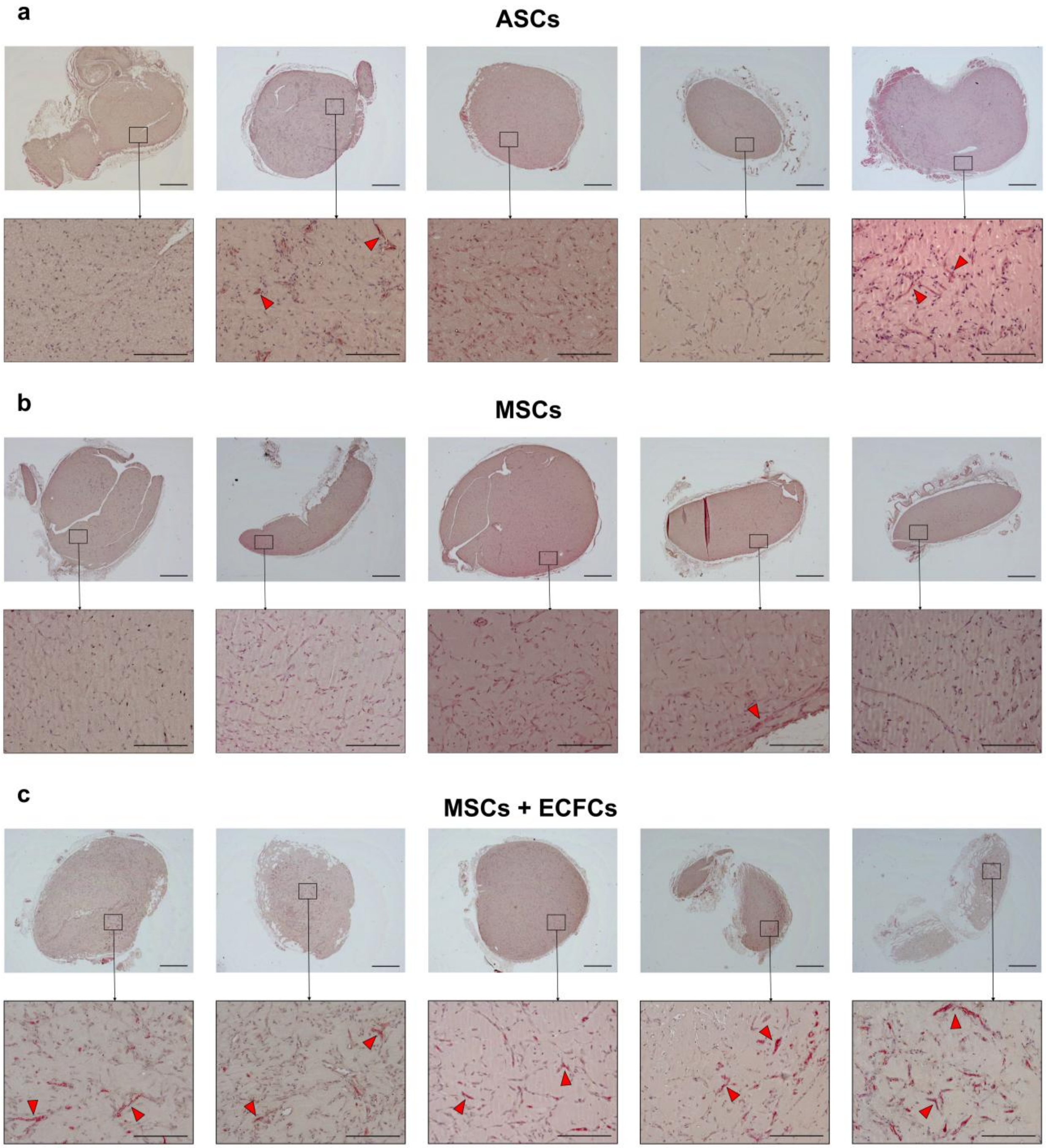

Supplementary Figure S3: Representative H&E staining of five implants from three groups (ASCs, MSCs, and ECFCs/MSCs). To obtain these implants, a) ASCs, b) MSCs, or c) the combination of ECFCs/MSCs were injected subcutaneously into nude mice. The implants were then excised, fixed, and sectioned for analysis. The figure shows sections from each group with a scale bar of 1 mm, along with magnified views of selected regions, displayed with a scale bar of 100 µm. Red arrowheads are used to indicate the location of blood vessels.

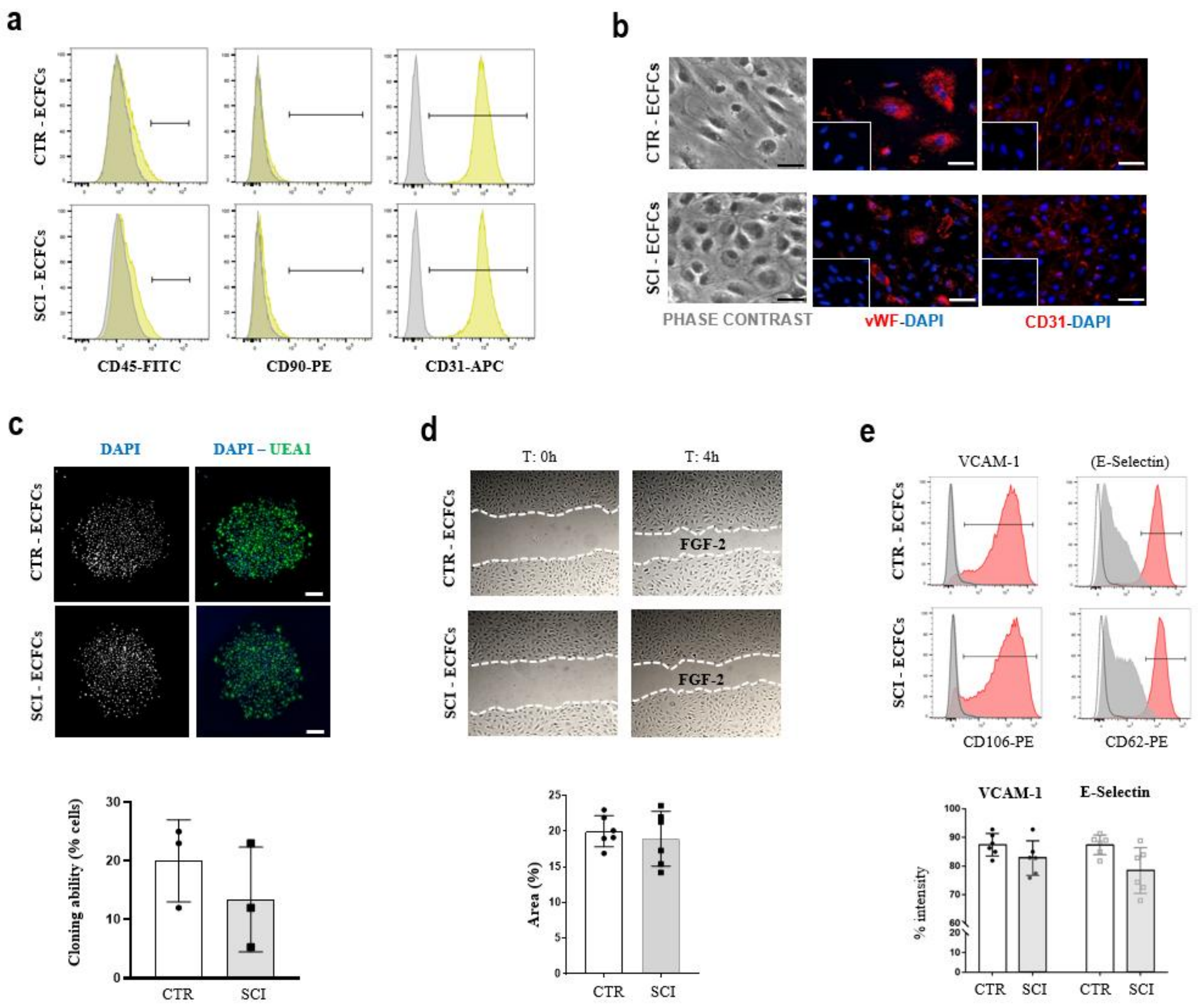

Supplementary Figure S4: **ECFCs Characterization and Functional Analysis**. **a)** Flow cytometry analysis of ECFCs for CD31, CD90, and CD45. Yellow line histograms represent cells stained with fluorescent antibodies. Unstained controls are shown in light blue, which overlap to produce a gray hue in the histograms. **b)** ECFCs visualized under phase contrast microscopy and indirect immunofluorescence showed positive staining for CD31 and vWF, both in red, while nuclei were counterstained with DAPI in blue. MSCs were used as a negative uptake control (inset). Scale bar: 50 µm. **c)** Comparative study of migration capacity in six samples per group (control [Ctr] and SCI patients). Migration was measured in response to FGF-2 (1 ng/mL). Representative micrographs show scratched ECFCs after 4 h in basal and FGF-2-containing medium (dashed lines delineate the scratch). Scratch size was quantified at 4 h as a percentage of the original size. No significant differences were observed between groups. **d)** Up-regulation of VCAM-1 and E-selectin in response to TNF-α. Representative flow cytometry histogram showing surface expression of VCAM-1 and E-selectin in ECFCs. Unstained cells are shown in black (dotted line), VCAM-1/E-selectin-stained cells without stimulation appear in gray (filled histogram), and cells stained after TNF-α stimulation (10 ng/mL, 4 h) are shown in red. The x-axis represents fluorescence intensity (log scale), and the y-axis indicates relative cell counts. TNF-α stimulation induced a marked upregulation of adhesion molecule expression, as evidenced by the rightward shift in the fluorescence intensity peak. No significant differences were observed. **e)** Clonogenic capacity of ECFCs was evaluated by seeding the cells at clonal density. Representative images (n = 3 per group) show colony formation in ECFCs derived from both SCI and CTR subjects, with two representative colonies shown for each group. scale bars: 0.5 mm. To quantify clonal ability, all colonies from each subject were counted, and the percentage of clonogenic cells was calculated. The bar graph shows the mean values per group. No significant differences were observed between SCI and CTR samples.

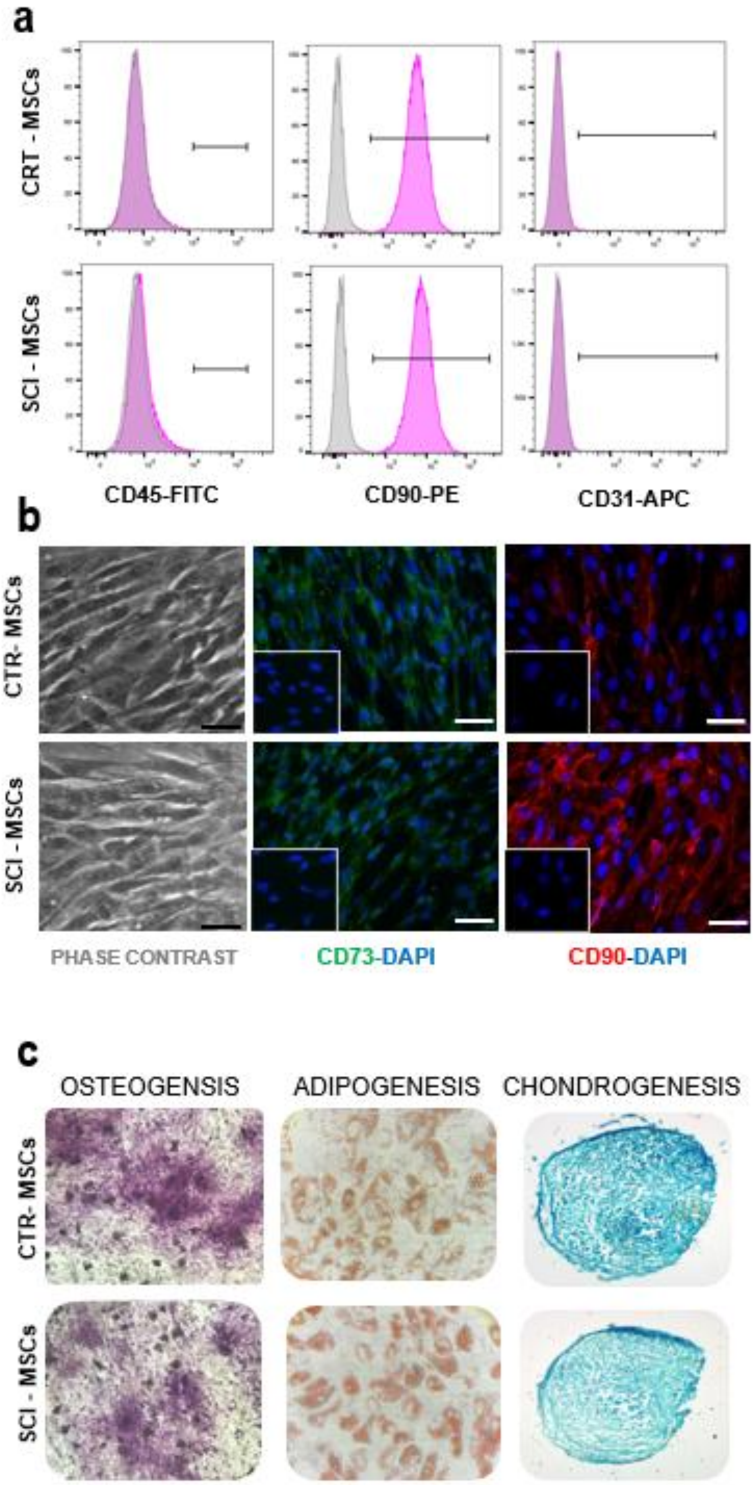

Supplementary Figure S5: **MSCs Characterization and Functional Analysis**. (a) Flow cytometry analysis of MSCs for CD31, CD90, and CD45. Pink line histograms represent cells stained with fluorescent antibodies. Unstained controls are shown in light blue. (b) MSCs visualized under phase contrast microscopy and indirect immunofluorescence showed positive staining for CD90 (in green) and CD73 (in red), while nuclei were counterstained with DAPI in blue. ECFCs were used as a negative uptake control (inset). Scale bar: 50 µm. (c) Multilineage differentiation of MSCs in vitro. Osteogenic differentiation was demonstrated by alkaline phosphatase staining. Chondrogenic differentiation was identified in pellet cultures through the presence of glycosaminoglycans, detected by Alcian blue staining. Adipogenic differentiation was confirmed by oil red O staining.
